# Left atrial remodeling and voltage-guided ablation outcome in persistent atrial fibrillation patients according to CHA_2_DS_2_-VASc score

**DOI:** 10.1186/s12872-024-04009-4

**Published:** 2024-07-08

**Authors:** Halim Marzak, Gabrielle Gennesseaux, Justine Hammann, Romain Ringele, Simon Fitouchi, François Severac, Thomas Cardi, Mohamad Kanso, Alexandre Schatz, Patrick Ohlmann, Olivier Morel, Laurence Jesel

**Affiliations:** 1 Department of Cardiovascular Medicine, Nouvel Hôpital Civil, Strasbourg University Hospital, Strasbourg, France; 2 Public Health Service, Groupe Méthodes en Recherche Clinique (GMRC), Strasbourg University Hospital, Strasbourg, France; 3 INSERM (French National Institute of Health and Medical Research), UMR 1260, FMTS, Regenerative Nanomedicine, Strasbourg, France

**Keywords:** Atrial fibrillation, CHAD_2_DS_2_-VASc score, Catheter ablation, Low voltage zone, Atrial bipolar voltage, Bipolar voltage map, Atrial remodeling

## Abstract

**Background:**

CHA_2_DS_2_-VASc score-related differences have been reported in atrial fibrotic remodeling and prognosis of atrial fibrillation (AF) patients after ablation. There are currently no data on the efficacy of low voltage zone (LVZ)-guided ablation in persistent AF patients according to CHA_2_DS_2_-VASc score. We assessed in a cohort of persistent AF patients the extent of LVZ, the regional distribution of LA voltage and the outcome of LA voltage-guided substrate ablation in addition to PVI according to CHA_2_DS_2_-VASc score.

**Methods:**

138 consecutive persistent AF patients undergoing a first voltage-guided catheter ablation were enrolled. 58 patients with CHAD_2_DS_2_-VASc score ≥ 3 and 80 patients with CHAD_2_DS_2_-VASc score ≤ 2 were included. LA voltage maps were obtained using 3D-electroanatomical mapping system in sinus rhythm. LVZ was defined as < 0.5 mV.

**Results:**

In the high CHAD_2_DS_2_-VASc score group, LA voltage was lower (1.5 [1.1–2.5] vs. 2.3 [1.5–2.8] mV, *p* = 0.02) and LVZs were more frequently identified (40% vs. 18%), *p* < 0.01). Female with CHA_2_DS_2_-VASc score ≥ 3 (*p* = 0.031), LA indexed volume (*p* = 0.009) and P-wave duration ≥ 150 ms (*p* = 0.001) were predictors of LVZ. After a 36-month follow-up, atrial arrhythmia-free survival was similar between the two groups (logrank test, *P* = 0.676).

**Conclusions:**

AF patients with CHAD_2_DS_2_-VASc score ≥ 3 display more LA substrate remodeling with lower voltage and more LVZs compared with those with CHAD_2_DS_2_-VASc score ≤ 2. Despite this atrial remodeling, they had similar and favorable 36 months results after one single procedure. Unlike male with CHAD_2_DS_2_-VASc score ≥ 3, female with CHAD_2_DS_2_-VASc score ≥ 3 was predictor of LVZ occurrence.

**Supplementary Information:**

The online version contains supplementary material available at 10.1186/s12872-024-04009-4.

## Background

Atrial fibrillation (AF), the most common cardiac arrhythmia is associated with an increased risk of complications such as thromboembolic event, heart failure and death [1, 2]. The CHA_2_DS_2−_VASc score is used to predict thromboembolic risk in non-valvular-AF patients and guide anticoagulation decision in clinical practice [3]. 

Several studies evidenced that CHA_2_DS_2_-VASc score was clearly associated with AF recurrence in patients undergoing AF catheter ablation (CA) and that it could also predict recurrence after CA [4–6]. As described by Chao[7], the patients with CHA_2_DS_2_-VASc score ≥ 3 and left atrial (LA) dimension ≥ 44 mm had all recurrence within the year after the initial CA [5–8]. LA volume and endocardial voltage seem also to be associated with CHA_2_DS_2_-VASc score and the risk of stroke. CA of AF has become an effective and first line therapy with increasing indications. Arrhythmia recurrence after persistent AF ablation is still a major issue. After pulmonary veins isolation, the optimal ablation targets are still under debate. Pulmonary vein isolation (PVI) in combination with LVZ-guided ablation could provide better results in persistent AF often associated with long-term 50% atrial arrhythmia (AA) recurrence [9]. 

Some studies investigated LA substrate remodeling according to CHA_2_DS_2_-VASc score [10, 11]. However, data on LVZ assessment by atrial region according to CHA_2_DS_2_-VASc score are currently scarce [12]. 

The purpose of our study was to assess in a cohort of persistent AF patients the extent of LVZ and the regional distribution of LA bipolar voltage according to CHA_2_DS_2_-VASc score. We also aimed to evaluate the outcome of LA voltage-guided substrate ablation in addition to PVI and to analyze predictive factors of LVZ and AF recurrence after CA.

## Methods

### Study population

**Fig. 1:**
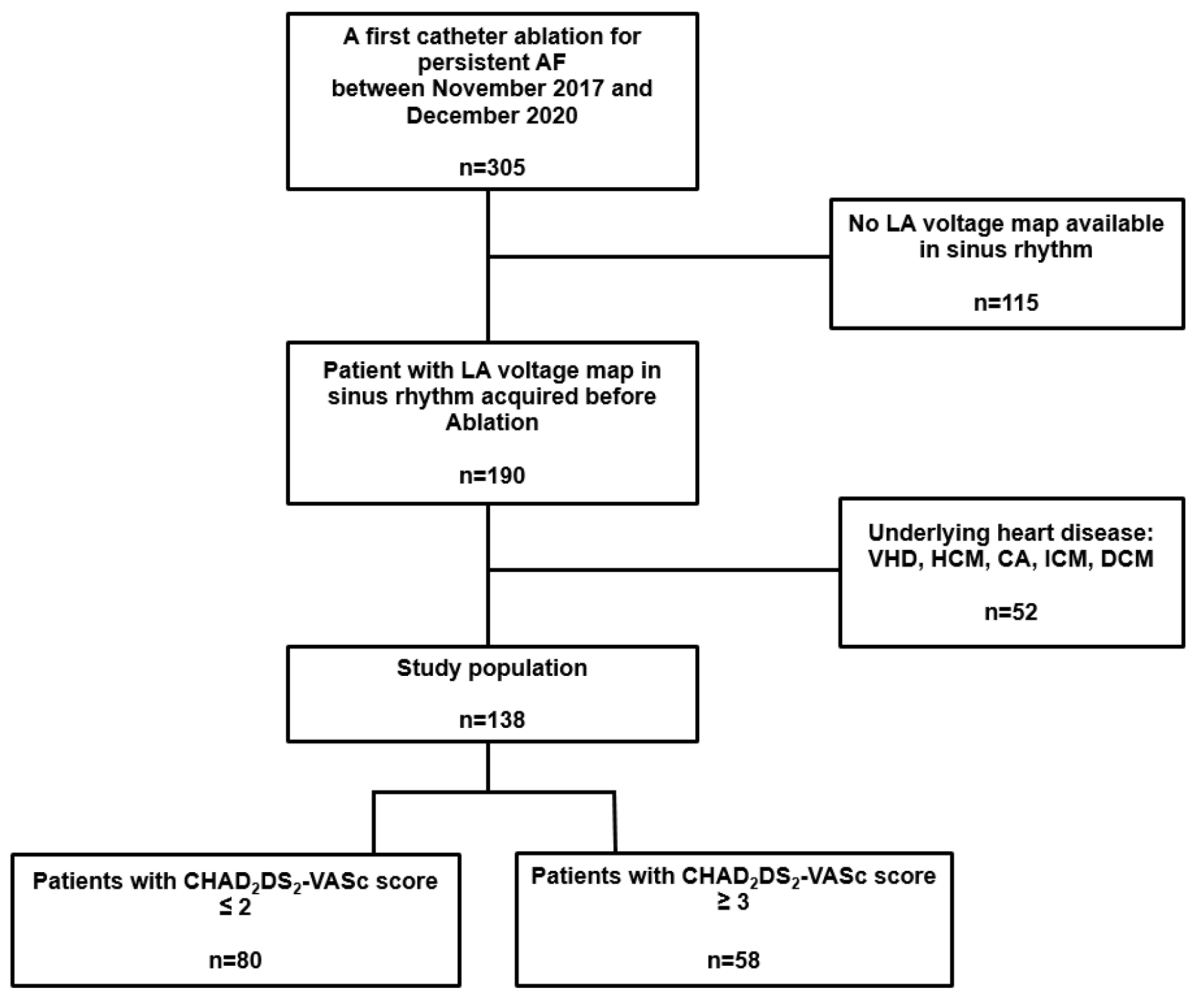
Flow Chart of the Study AF: atrial fibrillation; LA: left atrial; VHD: valvular heart disease; HCM: hypertrophic cardiomyopathy; CA: cardiac amyloidosis: ICM: ischemic cardiomyopathy; DCM: dilated cardiomyopathy

Between November 2017 and December 2020, 190 patients undergoing in our institution a first CA for persistent AF with LA voltage maps in sinus rhythm (SR) were retrospectively included. 138 patients were finally enrolled after excluding patients with structural heart disease (Fig. 1) which was defined by a previous diagnosis of ischemic heart disease, valve dysfunction (≥ moderate), or primary myocardial structural disease, including dilated or hypertrophic cardiomyopathies.

138 patients were divided into 2 groups according to the CHAD_2_DS_2_-VASc score cut-off of 3 corresponding to a high score. 58 patients with CHAD_2_DS_2_-VASc score ≥ 3 and 80 patients with CHAD_2_DS_2_-VASc score ≤ 2 were identified.

The CHAD_2_DS_2_-VASc score cut-off of 3, corresponding to a high score was chosen based on the study of Kiedrowicz [11]. This author reported that a CHA_2_DS_2_-VASc ≥ 3 score predicted the presence of LVZ in a long-standing persistent AF population.

Patient demographics and baseline clinical characteristics were collected, including sex, age, medical history, cardiovascular risk factors, medications, echocardiographic parameters, and electrocardiogram results at the time of admission and after follow-up. The study protocol was approved by the institutional review board of Strasbourg University (CE-2023-113). All patients gave their written informed consent for the ablation and their participation in this study.

### Procedural preparation

Patients were efficiently anticoagulated for at least 3 weeks. The antiarrhythmic drugs (AADs) were stopped for ≥ 5 half-lives before procedure. Amiodarone was discontinued three weeks before. CA procedures were performed under general anesthesia and three-dimensional electro-anatomical mapping (3D-EAM) system (CARTO 3, Biosense Webster, Diamond Bar, CA, USA and a multipolar mapping catheter PentaRay^®^ (Biosense Webster, Diamond Bar, CA, USA).

A transoesophagal echocardiography was used both to exclude any LA thrombi, especially in the left atrial appendage (LAA), and to guide the transseptal puncture. A decapolar catheter (steerable diagnostic catheter, Biosense Webster, Diamond Bar, CA, USA) was inserted in the coronary sinus (CS) for LA activity recording and stimulation. Two transseptal sheaths (SLO™ and/or Agilis™, Abott) were introduced into the LA via the femoral veins. A temperature probe was inserted into the esophagus for temperature monitoring. An activated clotting time was maintained between 250 and 350s during the procedure.

### LA voltage mapping

LA endocardial voltage maps were obtained in SR prior to CA. For patients in AF, an electrical cardioversion was achieved to restore the SR first. Endocardial contact during point acquisition was validated by a stable contact signal for > 2 beats. All points recorded in SR were analyzed to exclude mechanically induced premature beats.

For all patients, tissue proximity indication (TPI) was activated during LA mapping with the multipolar catheter. In rare cases, to eliminate doubt of a false low voltage area resulting from a difficult contact between the multipolar catheter and LA tissue, few regions were reanalyzed carefully point by point with a 4-mm irrigated contact-force ablation catheter (ThermoCool^®^ SmartTouch^®^, Biosense Webster, Diamond Bar, CA, USA or Tacticath^®^, Abbott, St Paul, MN, USA) to avoid any mistake. We only did that to confirm certain normally-volted regions. The evaluation of all LVZ had been done only with the multipolar catheter. LA was divided into six atrial regions: anterior, septal, posterior, inferior, lateral, and LAA. The roof was part of the anterior wall, as previously described [13]. Points were acquired in each region. The bipolar voltage amplitude was recorded for each point and within each individual region. The median LA bipolar voltage measurements for each specific region were calculated in all patients. The LA intracavitary volume (LAIV) excluding LAA was obtained after anatomic reconstruction for each patient and expressed in ml. LA intracavitary volume index (LAIVI), expressed in ml.m[2], meant LAIV indexed to the body surface.

LVZs were defined as sites of > 3 adjacent low-voltage points with a bipolar peak-to-peak voltage amplitude of < 0.5 mV[14, 15] and covering > 5% of the LA surface area (LVZ surface/LA surface > 5%, excluding the pulmonary venous (PV) antral region, LAA orifice, and mitral valve). This threshold value corresponds to the lowest degree of atrial fibrosis detected using LGE-MRI [15]. LVZ extent was classified into stage I (no or discrete LVZ, ≤ 5%), II (mild, > 5 to ≤ 20%), III (moderate, > 20 to ≤ 35%), or stage IV (severe, > 35%) according to the UTAH fibrosis classification [16]. Each region involving a LVZ was defined as a low-voltage region. The surface areas (cm^2^) of each atrial region and of the LVZ within each region were measured using the software 3D-EAM.

### Catheter ablation procedure

PVI was performed using a 4-mm irrigated contact-force ablation catheter to deliver point by point contiguous lesions with a target temperature of 43 °C, infusion rate of 17 ml/mn, and maximal power limit of 35 W (20–25 W for the posterior wall and 30–35 W for the anterior wall). The endpoint of PV isolation was a bidirectional conduction block between the LA and PVs.

For stage I patients, only PVI was performed, whereas an additional LVZ-guided ablation was carried out for stage II-IV patients. LVZ homogenization or isolation was performed by radiofrequency ablation in patients with a mild or moderate LVZ. The end point for LVZ homogenization was reached with significant reduction in local atrial electrograms and loss of local atrial capture.

Linear ablation across LVZ was performed when the LVZ ablation area could be considered as a critical isthmus site for potential macro-reentrant tachycardia. Linear lesions were also performed to isolate large LVZs from the rest of the healthy atrium, such as a posterior box by a roof line and an inferoposterior line. Atrial burst pacing to refractoriness was conducted from the proximal CS to try to induce any tachycardia. Inductible atrial tachycardias (AT) were ablated with AT termination without reinducibility. In the case of induced AF, no additional ablation was performed.

### Post ablation follow-up

All patients continued their AADs for at least 3 months to prevent early atrial arrhythmia (AA) recurrences. Patients were seen at 3, 6, and 12 months and every 6 months by their cardiologist. At each visit, a 12-lead electrocardiography (ECG) and a routine 24-hour ECG Holter monitoring were recorded to detect any AA recurrence defined by any documented AF, atrial flutter and AT lasting for over 30 s. Arrhythmic episodes occurring within the first 3 months (blanking period) were not counted in the evaluation of final success rates. In the absence of AA recurrence, AADs were gradually discontinued between 3 and 6 months post-ablation at the physician’s discretion. Patients with AA recurrence were encouraged to undergo a repeat AF catheter ablation after the 3-month blanking period.

### Statistical analysis

Continuous variables are expressed as means with standard deviations for normally distributed data. The variables with a non-normally distribution are presented as medians and interquartile ranges (25th -75th interquartile range). Differences of continuous variables were analyzed for statistical significance using the Student t test or the Wilcoxon test, depending on data distribution.

Categorical variables are given as absolute values and percentages. Statistical differences of categorical variables between the two groups were tested using the chi-square test or Fischer’s exact test. The Shapiro-Wilk test was utilized to determine the Gaussian distribution for each quantitative variable.

Kaplan-Meier survival curves were made in each group to analyze AAs recurrence rate after one AF ablation procedure. Log-rank test with Bonferroni correction was used to compare the two groups.

Binominal logistic regression was applied to calculate the odds ratio and a 95% confidence interval of independent variables associated with LVZs and AF recurrences. Variables selected for testing in the multivariate analysis were those with *p* < 0.10 in the univariate analysis.

Receiver operating characteristic (ROC) curves were calculated to assess the area under the curve (AUC), sensitivity and specificity of female with CHA_2_DS_2_-VASc score ≥ 3, male with CHA_2_DS_2_-VASc score ≥ 3 and CHA_2_DS_2_-VASc score ≥ 3 for LA LVZ with cut-off ≥ 5%.

Another ROC curve was performed to assess the performance of the LA indexed volume for the prediction of LVZ. The optimal threshold was identified using the Youden index.

All statistical analyses were performed using SPSS statistical software, version 23.0 (IBMCorp.). A two-tailed *p* value of < 0.05 was considered statistically significant.

## Results

### Baseline characteristics

The study population included 138 patients with persistent AF, 80 patients with CHAD_2_DS_2_-VASc score ≤ 2, and 58 patients with CHAD_2_DS_2_-VASc score ≥ 3. The baseline characteristics of the population are summarized in Table 1. The patients with high CHAD_2_DS_2_-VASc score as expected were significantly older with more cardiovascular risk factors (hypertension, diabetes mellitus, dyslipidemia) and more thromboembolic events. Half of the patients were female (28 (48.28%) vs. 13 (16.25%), *p* < 0.01). The HAS BLED score was also higher (1.9 (0.83) vs. 0.81 (0.71), *p* < 0.01).

**Table 1:** Baseline characteristics in persistent AF patients with CHAD_2_DS_2_-VASc scores ≤ 2 and ≥ 3

	CHAD_2_DS_2_-VASc score ≤ 2 (*n* = 80)	CHAD_2_DS_2_-VASc score ≥ 3 (*n* = 58)	*P* value
Age, years	62 (56.75-67)	70 (65–74)	**< 0.01**
Female sex, n (%)	13 (16.25%)	28 (48.28%)	**< 0.01**
CHA_2_DS_2_-VASc score, n	1.26 (0.72)	3.78 (0.9)	**< 0.01**
HAS BLED score, n	0.81 (0.71)	1.9 (0.83)	**< 0.01**
BMI, kg/m²	29.1 (26.9–32)	30.4 (25.9–32.9)	0.45
Hypertension, n (%)	35 (43.75%)	51 (87.93%)	**< 0.01**
Diabetes mellitus, n (%)	4 (5%)	22 (37.93%)	**< 0.01**
Dyslipidemia, n (%)	22 (27.5%)	28 (48.28%)	**0.02**
OSA, n (%)	23 (28.75%)	19 (32.76%)	0.75
eGFR, ml/min/1,73²	89 (74.75-95)	71 (60-89.5)	**< 0.01**
Coronary artery disease, n (%)	10 (12.5%)	5 (8.62%)	0.66
Time to treatment, days	702 (197–1531)	620 (240–1848)	0.96
Reported AF duration, months			0.09
< 3 months	62 (78.48%)	79.31% (46)	
≥ 3 to < 6 months	11 (13.92%)	4 (6.9%)	
≥ 6 to < 9 months	2 (2.53%)	7 (12.07%)	
≥ 9 to < 12 months	2 (2.53%)	0 (0%)	
≥ 12 months	2 (2.53%)	1 (1.72%)	
Thromboembolism, n (%)	2 (2.5%)	14 (24.14%)	**< 0.01**
P-wave duration ≥ 150 ms, n (%)	24 (30%)	31 (54.39%)	**< 0.01**
LVEF, % before ablation	61 (51–67)	58 (47-64.25)	0.26
Per-procedural LAIV excluding LAA, ml	132.5 (110-151.3)	130 (117.5–150)	0.87
Per-procedural LAIVI excluding LAA, ml.m^2^	62.6 (53.2–68.6)	64.8 (56–74)	0.13
Beta-blocker	65 (81.25%)	50 (86.21%)	0.59
ACEi/ARB	45 (56.25%)	41 (70.69%)	0.12
Aldosterone receptor antagonist	12 (15%)	17 (29.31%)	0.07

Data are presented as a value (with percentage) for categorical variables, median (25th − 75th percentile) or mean ± SD for quantitative variables. A two-tailed *p* value of < 0.05 was considered significant

Time to treatment = time from first clinical diagnosis of AF to ablation procedure

**Abbreviations**:

*AF = atrial fibrillation; BMI = body mass index; OSA = obstructive sleep apnea*; *eGFR = estimated glomerular filtration rate; LVEF = left ventricle ejection fraction; LAIV = left atrial intracavitary volume; LAIVI = left atrial intracavitary volume index; LAA = left atrial appendage; ACEi/ARB = angiotensin-convertinge enzyme inhibitor/*Angiotensin II receptor blocker

No difference could be observed between the two groups in term of AF duration, LA volumes, time to treatment and beta-blocker, ACEi/ARB and aldosterone receptor antagonist (ARA) use.

In the high CHAD_2_DS_2_-VASc score group, P-wave duration (PWD) ≥ 150ms was more frequent (31 (54.4%) vs. 24 (30%), *p* < 0.01) and kidney function was lower (71 [60-89.5] vs. 89 [74.75-95] ml/min/1,73², *p* < 0.01).

### Left atrial bipolar voltage assessment

The median number of total mapping points per patient was similar in the two groups (1277 [789–1953] vs. 1320 [995–1862] points, *p* = 0.85) (Table 2). The global LA bipolar voltage was lower in the high CHAD_2_DS_2_-VASc score group (1.5 [1.1–2.5] vs. 2.3 [1.5–2.8] mV, *p* = 0.02). The regional bipolar voltage amplitudes in this group were also lower in the anterior LA (1.4 [0.9–1.9] vs. 2 [1.3–2.7] mV, *p* < 0.01), posterior LA (1.59 [1-2.7] vs. 2.28 [1.4–3.1] mV, *p* = 0.02), and in the LAA (2.5 [1.5–3.4] vs. 3 [2-3.9] mV, *p* = 0.04). Septal, inferior and lateral bipolar voltage amplitudes were similar in the two groups.

**Table 2:** Median regional distribution of bipolar voltage amplitudes and low-voltage zones by distribution and extent in persistent AF patients with CHAD_2_DS_2_-VASc scores ≤ 2 and ≥ 3

	CHAD_2_DS_2_-VASc score ≤ 2 (*n* = 80)	CHAD_2_DS_2_-VASc score ≥ 3 (*n* = 58)	*P* value
Median total mapping points per patient	1277 [789–1953]	1320 [995–1862]	0.85
**Median and regional bipolar voltage amplitude, mV**			
Global left atrium	2.25 (1.49–2.79)	1.49 (1.1–2.48)	**0.02**
Anterior	1.97 (1.29–2.67)	1.39 (0.87–1.93)	**< 0.01**
Posterior	1.69 (1.02–2.34)	1.2 (0.86–2.04)	0.06
Inferior	2.28 (1.42–3.07)	1.59 (0.96–2.68)	**0.02**
Lateral	1.99 (1.14–2.57)	1.69 (1.12–2.64)	0.56
Left Atrial Appendage	2.23 (1.25–3.05)	2.1 (1.18–2.86)	0.7
**LVZ extent calculated as the percentage of LA surface area**			
No LVZ, n (%)	66 (82.5%)	35 (60.34%)	**< 0.01**
LVZ, n (%)	14 (17.5%)	23 (39.66%)	**< 0.01**
Mild LVZ, n (%)	5 (6.33%)	11 (18.97%)	**0.04**
Moderate LVZ, n (%)	7 (8.86%)	5 (8.62%)	1
Severe LVZ, n (%)	2 (2.53%)	7 (12.07%)	**0.04**
**Number of regional LVZs, n (%)**			
Anterior	16 (20.25%)	24 (41.38%)	**0.01**
Septal	18 (22.78%)	18 (31.03%)	0.37
Posterior	8 (10.13%)	12 (20.69%)	0.14
Inferior	1 (1.27%)	7 (12.07%)	**0.01**
Lateral	1 (1.27%)	3 (5.17%)	0.31
LAA	0 (0%)	6 (10.34%)	**< 0.01**

All data are presented as a value (percentage) for categorical variables or median (25th–75th percentile) for quantitative variables. A two-tailed *p* value of < 0.05 was considered significant

**Abbreviations**:

*AF = atrial fibrillation*; *LA = left atrium; LVZ = low voltage zone; LAA = left atrial appendage*

### Low-voltage zones assessment

LVZs were found in 27% of the whole cohort (Table 2). LVZ were more frequent in the high CHAD_2_DS_2_-VASc score group (39.7% vs. 17.5%, *p* < 0.01), especially in case of mild LVZ (19% vs. 6.3%, *p* = 0.04) and severe LVZ (12.1% vs. 2.5%, *p* = 0.04). When analyzing LVZs according to atrial region, patients with high CHAD_2_DS_2_-VASc score had more anterior LVZ (41.4% vs. 20.3%, *p* = 0.01), inferior LVZ (12.1% vs. 1.3%, *p* = 0.01) and LVZ in the LAA (10.3% vs. 0%, *p* < 0.01) compared with patients with low CHAD_2_DS_2_-VASc score.

Patients with LVZ, predominantly female (62% vs. 18%, *p* < 0.01) were older (70 [68–74] vs. 62 [56–68], *p* < 0.01) and presented with a mild altered kidney function (71 [63–80] vs. 88 [69–95], *p* < 0.01) compared to those without LVZ. Indexed to body surface or not, intracavitary LA volumes were increased (150 [120–160] vs. 129 [110–140] ml, *p* = 0.01) in patients with LVZ. P-wave duration ≥ 150 ms was more often observed in patients with LVZ (30 (83%) vs. 25 (25%), *p* < 0.01) (Supplemental Table 1).

### Ablation results

All PVs were successfully isolated during CA (Supplemental Table 2). PVI alone was performed in 73.2% (101) patients of the overall cohort, while the remaining 26.8% (37) had additional LVZ-guided ablation.

PVI alone was less frequently performed in patients with high CHAD_2_DS_2_-VASc score 60.3% (35) vs. 82.3% (65), *p* < 0.01). There was no difference between the two groups for linear ablation (20% [16] vs. 32.8% [17], *p* = 0.13) as for regional lines. There was also no difference between the two groups for CTI ablation before or during the procedure (17.2% vs. 20%, *p* = 0.85). The total RF duration was longer in patients with high CHAD_2_DS_2_-VASc score (32.3 [24.2–43.3] vs. 28.2 [23.7–34.3] minutes, *p* = 0.06) but the difference was not significant. The fluoroscopy time was similar between the two groups (22 [19.3–30] vs. 22.6 [17.6–29.4 ] minutes, *p* = 0.51).

Postprocedural complications occurred in 5.1% (7/138) of the overall cohort, mainly perivascular complication. No difference was observed between the two groups (Supplemental Table 2).

### Long-term clinical outcome after one AF ablation procedure

**Fig. 2:**
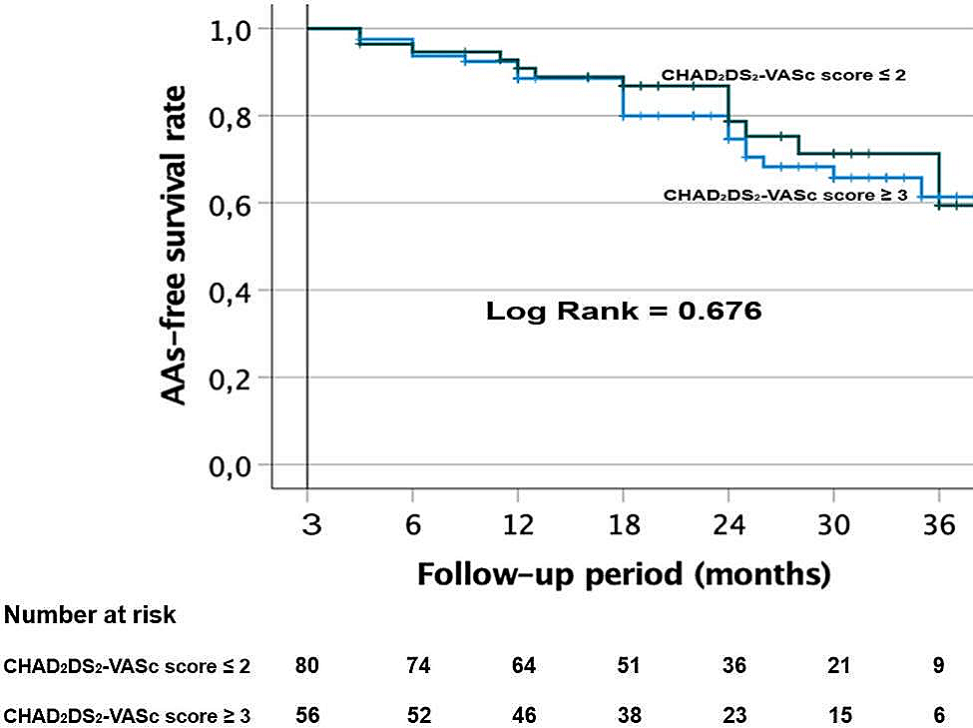
Kaplan-Meier survival curves showing the cumulative AF/AT recurrence-free survival rates in the patients with CHAD_2_DS_2_-VASc scores ≤ 2 and ≥ 3 after a single procedure AAs = atrial arrhythmias; AF = atrial fibrillation; AT = atrial tachycardia

After a follow-up period of 37.1 [33.7–40.4] months, there was no difference in the AA-free survival rate after one procedure between the two groups (log rank test, *p* = 0.676). In all, 90.9 ± 3.9% of patients with CHAD_2_DS_2_-VASc score ≥ 3 and 88.5 ± 3.6% of patients with CHAD_2_DS_2_-VASc score ≤ 2 remained free of AF/AT after 12 months (Fig. 2). At 24 months, 78.7 ± 6.2% of patients with high CHAD_2_DS_2_-VASc score and 74.6 ± 5.3% of patients with low CHAD_2_DS_2_-VASc score were free of AAs. At 36 months, 59.4 ± 9.9% of patients with high CHAD_2_DS_2_-VASc score and 61.4 ± 7.2% of patients with low CHAD_2_DS_2_-VASc score were free of AAs. In the whole cohort, AADs were discontinued in 74.6% (103/138) of the patients.

Among patients without AA recurrence at 36 months, AADs were discontinued in 79.5% (31/39) of the cohort, 64.3% (9/14) in patients with CHAD_2_DS_2_-VASc score ≥ 3 and 88% (22/25) in patients with CHAD_2_DS_2_-VASc score ≤ 2 (*p* = 0.109).

Finally, we observed no difference in AAs-free survival rate after one procedure between patients without LVZ who underwent PVI alone and those with LVZ who underwent PVI and additionnal LVZ ablation (log rank test, *P* = 0.972). 61% of patients with PVI alone and 59% of those with additionnal LVZ ablation remained free of AF/AT after 36 months (Supplemental Fig. 1).

### Predictors of low-voltage zone

To evaluate the predictive factors of LVZ, univariate and multivariate analysis were performed in the whole population. Female with CHA_2_DS_2_-VASc score ≥ 3, BMI, eGFR, LA indexed volume, time to treatment, AF duration > 6 months, and PWD ≥ 150 ms were selected as variables for a multivariate analysis. Female with CHA_2_DS_2_-VASc score ≥ 3 (OR 9.112, 95% CI 1.219–68.131, *p* = 0.031), LA indexed volume (OR 1.071, 95% CI 1.018–1.128, *p* = 0.009) and PWD ≥ 150 ms (OR 9.503, 95%CI 2.479–36.432, *p* = 0.001) were identified as independent predictors of LVZ (Table 3).

**Table 3:** Univariate and multivariate analyses for prediction of LVZ in the whole cohort

VARIABLE	Univariate Analysis			Multivariate Analysis		
	OR	95% CI	*P* Value	OR	95% CI	*P* Value
Female with CHA_2_DS_2_-VASc score ≥ 3	6.550	2.676–16.032	**< 0.001**	9.112	1.219–68.131	**0.031**
Male with CHA_2_DS_2_-VASc score ≥ 3	1.684	0.629–4.512	0.300	…	…	…
Dyslipidemia	0.924	0.424–2.016	0.843	…	…	…
OSA	1.867	0.770–4.526	0.167	…	…	…
BMI	0.886	0.814–0.965	**0.006**	0.856	0.700-1.048	0.133
eGFR	0.964	0.942–0.986	**0.002**	0.988	0.950–1.029	0.567
Coronary artery disease	0.527	0.174–1.599	0.258	…	…	…
LAIVI	1.089	1.044–1.135	**< 0.001**	1.071	1.018–1.128	**0.009**
Time to treatment	1.000	1.000-1.001	**0.059**	1.000	1.000-1.001	0.213
AF duration > 6 months	0.337	0.110–1.037	**0.058**	1.463	0.211–10.136	0.700
P-wave duration ≥ 150 ms	15.000	5.593–40.227	**< 0.001**	9.503	2.479–36.432	**0.001**

Data are presented as an odd ratio with 95% CI. A two-tailed *p* value < 0.05 was considered significant

Time to treatment = time from first clinical diagnosis of AF to ablation procedure

**Abbreviations**

LVZ; low voltage zone; OR = odds ratio; CI = confidence interval; OSA = obstructive sleep apnea; BMI = body mass index; eGFR = estimated glomerular filtration rate; LAIVI = left atrial intracavitary volume index; AF = atrial fibrillation

ROC analysis evidenced that the area under the curve (AUC) of female with CHA_2_DS_2_-VASc score ≥ 3, male with CHA_2_DS_2_-VASc score ≥ 3 and CHA_2_DS_2_-VASc score ≥ 3 to predict the occurrence of LA LVZ (cut-off: ≥5%) was 67 [56–78]%, 46 [35–56]%, and 63 [52–73]%, respectively. The sensitivity was 44.7%, 15.8%, and 60.5%, respectively. The specificity was 89%, 76%, and 65%, respectively (Supplemental Fig. 2).

LA indexed volume > 74 ml/m^2^ was the optimal cut-off value to predict the presence of LA LVZ with the highest Youden index at 1.46. The AUC was 78 [68–89] %. The sensitivity was 52% and the specificity was 84% (Supplemental Fig. 3^3^).

## Discussion

In the present study, we report that in persistent AF, patients with CHAD_2_DS_2_-VASc score ≥ 3 display more LA electrophysiological substrate remodeling with lower bipolar voltage and more LVZs, even though LA volumes were similar in both groups. Despite this significant fibrotic remodeling, patients with CHAD_2_DS_2_-VASc score ≥ 3 have a similar and favorable 36 months outcome after one single voltage-guided AF ablation. Unlike male with CHAD_2_DS_2_-VASc score ≥ 3, female with CHAD_2_DS_2_-VASc score ≥ 3, LA indexed volume and PWD ≥ 150 ms were independent predictors of LVZ occurrence.

Some publications assessed LA substrate remodeling according to CHA_2_DS_2_-VASc score [10, 11]. Müller showed that the CHA_2_DS_2_-VASc score was significantly higher in patients with higher extent of LA LVZ [10]. As for Kiedrowicz, the CHA_2_DS_2_-VASc ≥ 3 score predicted the presence of LVZ [11]. Data on LA substrate remodeling by atrial region according to CHA_2_DS_2_-VASc score are scarce. Park reported that global LA voltage amplitude as well as anterior and LAA voltage amplitudes were lower in patients with high CHAD_2_DS_2_-VASc score and non-valvular AF. In patients who experienced stroke, LA endocardial voltage was also lower than those without stroke [12]. We also evidenced a significant relationship with LA remodeling, CHAD_2_DS_2_-VASc score and stroke. We provided an information about LA remodeling with the regional distribution and extent of LVZ in persistent AF patients according to CHAD_2_DS_2_-VASc score. We evidenced that LVZ were more frequent in patients with high score especially for mild and severe LVZ. When studying LVZ by atrial region, patients with high CHAD_2_DS_2_-VASc score had both more anterior, inferior and LAA LVZ.

Some factors like hypertension, diabetes mellitus failed individually to predict LVZ in multivariate analysis while this abnormal atrial substrate attributable to hypertension[18] and diabetes mellitus[19], has been demonstrated to be involved in different experimental animal models. Some authors have also reported the association between LVZ, age[13, 17] and female gender [17, 20]. In our study, we could observe that the combination of these factors expressed as a high CHAD_2_DS_2_-VASc score ≥ 3 was predictive for the presence of LVZs. In a previous study, a mean risk score of 2.5–2.6 was an independent predictor for LVZ [21]. CHAD_2_DS_2_-VASc score is interesting because it pools several cardiovascular risk factors. Most of them like diabetes and hypertension share complex interplays with inflammation, senescence and endothelial dysfunction and could act as an amplification loop. In this context, renin angiotensin system is also known to be activated and leads among others to fibrosis through TGFß pathway activation. Inflammation with leucocytes infiltration and oxidative stress with excessive ROS production are great inductors of metalloproteinase expression and other mechanisms inducing extracellular matrix remodeling leading to final fibrosis infiltrating atrial tissue. LVZ attest to this final pathway. It is known that in persistent AF, atrial substrate remodeling can preexist before AF onset. Rovaris G et al. observed in a cohort of 2410 patients without previous AF and implanted with a holter that occurrence of AF episodes increased with CHA_2_DS_2_-VASc after a follow-up of 24 months. The association was even stronger with CHA_2_DS_2_-VASc ≥ 5 and long episodes of AF [22]. 

Interestingly, we also observed that patients with high CHA_2_DS_2_-VASc score presented a mild altered kidney function compared to those with low score although eGFR remained superior to 60 ml/min/1.73m^2^. Several studies found that renal dysfunction, defined as eGFR < 60 ml/min/1.73m^2^ (CKD) was an independent predictor for both the presence of LVZs and recurrence after AF ablation [23]. Renal function also reflects vascular disease.

CHAD_2_DS_2_-VASc score, AF and stroke are strongly associated but the causal relationship is complex. It has been shown that AF was not necessarily a direct cause of stroke [24]. However, it is also well established that the risk of stroke in patients with AF increases with high CHAD_2_DS_2_-VASc score [25, 26]. In our study, we observed that the patients with CHAD_2_DS_2_-VASc score ≥ 3 were significantly associated with both LA substrate remodeling and thromboembolic events. Park showed for the first time that LA electroanatomical remodelling had significant relationship with events of stroke in patients with non-valvular AF[12], sharing this same observation with other studies [10, 27]. As observed in our study, Kim also identified that female sex, particularly when their CHAD_2_DS_2_-VASc score were ≥ 3 was associated with extensive LVZ [28]. 

We also reported in our cohort of persistent AF that PWD ≥ 150ms and LA volume were predictive of LVZ. Jadidi also found that PWD of ≥ 150ms identified patients with advanced LA LVZ who are at high risk for arrhythmia recurrence after alone PVI [29]. In addition, a recent metaanalysis concluded that a PWD > 149.5ms in SR was predictive of increased arrhythmia recurrences in patients with paroxysmal AF after PVI [30]. LA enlargement is also well known to be associated with the presence of LVZ [13]. Park observed that LA volume was significantly higher in patients with high CHA_2_DS_2_-VASc score whereas we could not observe any difference [12]. Only 38% of the patients presented with persistent AF in his study. In our cohort, all AF were persistent and we could identify that LA index volume was predictive of LVZ presence.

With the current increase in CA activity and the broadening of indications, one-shot systems are of interest for a fast and safe ablation. Nevertheless, PVI alone is not enough in case of important LA remodeling and LVZ. CHAD_2_DS_2_-VASc ≥ 3, LA indexed volume and P-wave duration may be good indicators of the presence of LVZ and could help the operator to optimize the choice of the catheter type.

Several studies reported that the increase in CHAD_2_DS_2_-VASc was significantly correlated with a poor outcome after AF ablation[4, 5, 7] particularly when CHA_2_DS_2_-VASc score was > 3 [5, 7]. The same has been observed in case of hypertension or diabetes alone [31, 32]. LVZs are also known to be a powerful predictor of recurrence after AF ablation [9]. Interestingly, in our study, the first to assess the results of voltage-guided ablation in persistent AF patients according to the CHAD_2_DS_2_-VASc score, we showed that the results of ablation were similar in the group with low and high CHAD_2_DS_2_-VASc score after 36 months of follow-up. Nevertheless, we can observe that antiarrhythmic drugs were not systematically stopped after CA particularly in the high CHAD_2_DS_2_-VASc which could disrupt the evaluation of this ablation strategy. However, these results are encouraging for a tailored ablation strategy in addition to PVI in persistent AF and high CHAD_2_DS_2_-VASc score patients.

This study has several limitations. It is a single-center observational non-randomized study with a retrospective design and long-term analysis. The number of patients in this retrospective analysis is limited that may influence the results of our work. Thus, larger patient populations are needed to strengthen the conclusions drawn.

However, only two operators carried out the procedures with a similar protocol to limit bias. In addition, a great amount of points were also collected and analyzed with high-density catheters during 3D mapping for a more rapid and a better resolution, particularly for LVZ assessment. The CA results were assessed until 36 months in postablation for a large part of our population with a median follow-up of 37.1 [33.7–40.4] months. A longer follow-up period could provide valuable insights, particularly for evaluating the CA outcomes for the repeated procedure. In addition, AADs discontinuation could not be obtained for the whole cohort because the follow-up was performed by the patients’ individual cardiologists. It could have influenced the results of ablation. Our study is among the first to assess the results of LVZ-guided ablation in persistent AF patients depending on the CHAD_2_DS_2_-VASc score. Further multicenter randomized studies are mandatory to assess the long-term follow-up after voltage-guided AF ablation according to the CHAD_2_DS_2_-VASc score.

## Conclusions

Persistent AF patients with high CHAD_2_DS_2_-VASc score displayed more LA substrate remodeling with lower bipolar voltage and more frequent LVZs. Despite this extensive fibrotic remodeling, they had a similar and favorable 36 months outcome after one single voltage-guided AF ablation compared to those with low CHAD_2_DS_2_-VA score. Unlike male with CHAD_2_DS_2_-VASc score ≥ 3, female with CHAD_2_DS_2_-VASc score ≥ 3, LA indexed volume and PWD ≥ 150 ms were independent predictors for the presence of LVZ in the whole cohort. These results highlight the multifactorial nature of LVZ development and the complex interplay between LA remodeling and the different risk factors composing the CHAD_2_DS_2_-VASc score.

### Electronic supplementary material

Below is the link to the electronic supplementary material.

Supplementary Material 1

## Data Availability

The datasets generated during and/or analyzed during the current study are not publicly available due to their containing information that could compromise the privacy of patients but are available from the corresponding author on reasonable request.

## Abbreviations

AAs: atrial arrhythmias
AADs: antiarrhythmic drugs
AF: atrial fibrillation
AT: atrial tachycardia
ACEi/ARB: angiotensin-converting enzyme inhibitor/Angiotensin II receptor blocker
AUC: area under the curve
BMI: body mass index
CA: catheter ablation
CA: cardiac amyloidosis
CI: confidence interval
CS: coronary sinus
CTI: cavo-tricuspid isthmus
DCM: dilated cardiomyopathy
3D-EAM: three-dimensional electro-anatomical mapping
ECG: 12-lead electrocardiography
eGFR: estimated glomerular filtration rate
HCM: hypertrophic cardiomyopathy
ICM: ischemic cardiomyopathy
LA: left atrium
LAA: left atrial appendage
LAIV: left atrial intracavitary volume
LAIVI: left atrial intracavitary volume index
LGE-MRI: late gadolinium enhancement - magnetic resonance imaging
LVEF: left ventricle ejection fraction
LVZ: low voltage zone
Min: minute
OR: odds ratio
OSA: obstructive sleep apnea
PV: pulmonary venous
PVI: pulmonary vein isolation
PWD: P-wave duration
RF: radiofrequency
ROC: receiver operating characteristic
SR: sinus rhythm
TPI: tissue proximity indication
VHD: valvular heart disease
