# Supplementary material for: Left atrial remodeling and voltage-guided ablation outcome in persistent atrial fibrillation patients according to CHA2DS2-VASc score
Source: BMC Cardiovasc Disord. 2024 Jul 8;24:347. doi: 10.1186/s12872-024-04009-4 (PMC11229227; doi:10.1186/s12872-024-04009-4)
Supplement: Supplementary file 1 — Supplementary Material 1 [file 12872_2024_4009_MOESM1_ESM.doc]

**Supplemental Table 1.** Baseline Characteristics in persistent AF patients according to presence or not of LVZ.

|  | **No LVZ (n=100)** | **LVZ (n=37)** | **P value** |
| --- | --- | --- | --- |
| Age, years | 62 (56-68) | 70 (68-74) | **<0.01** |
| Female sex, n (%) | 18 (18%) | 23 (62%) | **<0.01** |
| CHA_2_DS_2_-VASc score, n | 2 [1-3] | 3 [2-4] | **<0.01** |
| HAS BLED score, n | 1 [0-2] | 2 [1-2] | **<0.01** |
| BMI, kg/m² | 29 [26-32] | 30.4 [26-33] | 0.4 |
| Hypertension, n (%) | 58 (58%) | 27 (73%) | 0.16 |
| Diabetes mellitus, n (%) | 18 (18%) | 8 (22%) | 0.81 |
| Dyslipidemia, n (%) | 36 (36%) | 14 (38%) | 1 |
| OSA, n (%) | 34 (34%) | 8 (22%) | 0.24 |
| eGFR, mL/min/1,73² | 88 [69-95] | 71 [63-80] | **<0.01** |
| Coronary artery disease, n (%) | 9 (9%) | 6 (16%) | 0.23 |
| Time to treatment, days | 487 [183-1403] | 1179 [398-2282] | **0.03** |
| Reported AF duration, months |  |  | 0.41 |
| < 3 months | 81 (82%) | 27 (73%) |  |
| ≥ 3 to < 6 months | 11 (11%) | 4 (11%) |  |
| ≥ 6 to < 9 months | 4 (4%) | 4 (11%) |  |
| ≥ 9 to < 12 months | 1 (1%) | 1 (2.5%) |  |
| ≥ 12 months | 2 (2%) | 1 (2.5%) |  |
| Thromboembolism, n (%) | 11 (11%) | 5 (14%) | 0.77 |
| P-wave duration ≥ 150 ms, n (%) | 25 (25%) | 30 (83%) | **<0.01** |
| LVEF, %, before ablation | 58 [50-65] | 60 [52-70] | 0.19 |
| Per-procedural LAIV excluding LAA, ml | 129 [110-140] | 150 [120-160] | **0.01** |
| Per-procedural LAIVI excluding LAA, ml.m^2^ | 61 (52-66) | 75 (64-85) | **<0.01** |
| Beta-blocker | 86 (86%) | 28 (76%) | 0.24 |
| ACEi/ARB | 64 (64%) | 21 (57%) | 0.56 |
| Aldosterone receptor antagonist | 21 (21%) | 8 (22%) | 1 |

Data are presented as a value (with percentage) for categorical variables, median (25^th^–75^th^ percentile) or mean±SD for quantitative variables. A two-tailed p value of <0.05 was considered significant. Time to treatment = time from first clinical diagnosis of AF to ablation procedure**.**

**Abbreviations:** *AF = atrial fibrillation*; BMI = body mass index; OSA = *obstructive sleep* apnea; eGFR = estimated glomerular filtration rate; LVEF = left ventricle ejection fraction; LAIV = left atrial intracavitary volume; LAIVI = left atrial intracavitary volume index; LAA = left atrial appendage; ACEi/ARB = angiotensin-convertinge enzyme inhibitor/Angiotensin II receptor blocker.

**Supplemental Table 2.** Procedural data related characteristics.

| **VARIABLE** | **CHAD_2_DS_2_-VASc score ≤ 2 (n=80)** | **CHAD_2_DS_2_-VASc score ≥ 3 (n=58)** | **P value** |
| --- | --- | --- | --- |
| Fluoroscopic time, min | 22.6 [17.6-29.4] | 22 [19.3-30] | 0.51 |
| Total RF duration, min | 28.2 [23.7-34.3] | 32.3 [24.2-43.3] | 0.06 |
| AF on admission before procedure | 23 (28.8%) | 20 (34.5%) | 0.6 |
| Only PVI | 65 (82.3%) | 35 (60.3%) | **<0.01** |
| LVZ ablation | 14 (17.7%) | 23 (39.7%) | **<0.01** |
| Linear ablation | 16 (20%) | 19 (32.8%) | 0.13 |
| Anterior line | 8 (10%) | 12 (20.7%) | 0.13 |
| Posterior line | 8 (10%) | 9 (15.5%) | 0.48 |
| Roof line | 6 (7.5%) | 10 (17.2%) | 0.3 |
| Septal line | 9 (15.5%) | 12 (15%) | 1 |
| Mitral line | 0 (0%) | 1 (1.7%) | 0.42 |
| CTI ablation before or during procedure | 16 (20%) | 10 (17.2%) | 0.85 |
| Complications | 2 (2.5%) | 5 (8.6%) | 0.13 |
| Scarpa’s hematoma | 1 (1.25%) | 3 (5.17%) | 0.31 |
| Stroke | 0 (0%) | 0 (0%) | *** |
| Cardiac tamponade | 0 (0%) | 1 (1.72%) | 0.42 |

Data are presented as a value (with percentage) for categorical variables, median (25^th^-75^th^ percentile) for quantitative variables. A two-tailed p value of <0.05 was considered significant. *AF = atrial fibrillation; Min = minute; RF = radiofrequency; PVI = pulmonary vein isolation; LVZ = low voltage zone; CTI = cavo-tricuspid isthmus ablation*.

**Supplemental Figure 1.** Kaplan-Meier survival curves showing the cumulative AAs recurrence-free survival rates between patients without LVZ (who underwent PVI alone) and those with LVZ (who underwent PVI and additionnal LVZ ablation) after a single ablation procedure.


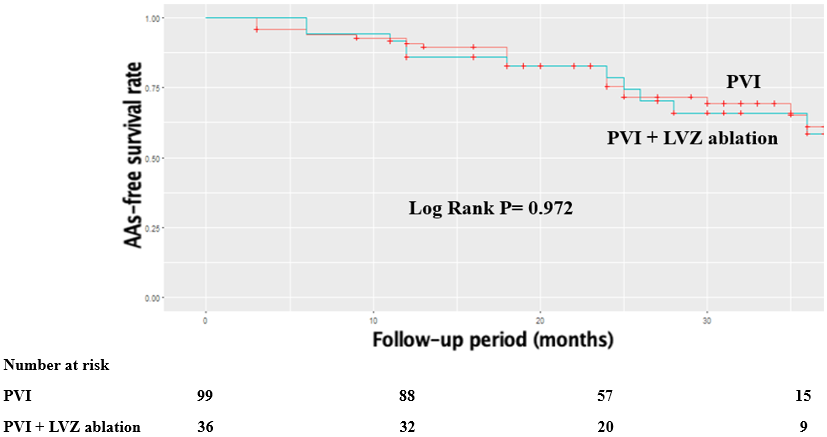


**Abbreviations:** *AAs, atrial arrhythmias; PVI, pulmonary vein isolation; LVZ, low-voltage zone.*

**Supplemental Figure 2.** Receiver operating charecteristic curves for female with CHA_2_DS_2_-VASc score ≥3, male with CHA_2_DS_2_-VASc score ≥3 and CHA_2_DS_2_-VASc score ≥3 as a predictor of LA LVZ (cut-off: ≥5%)


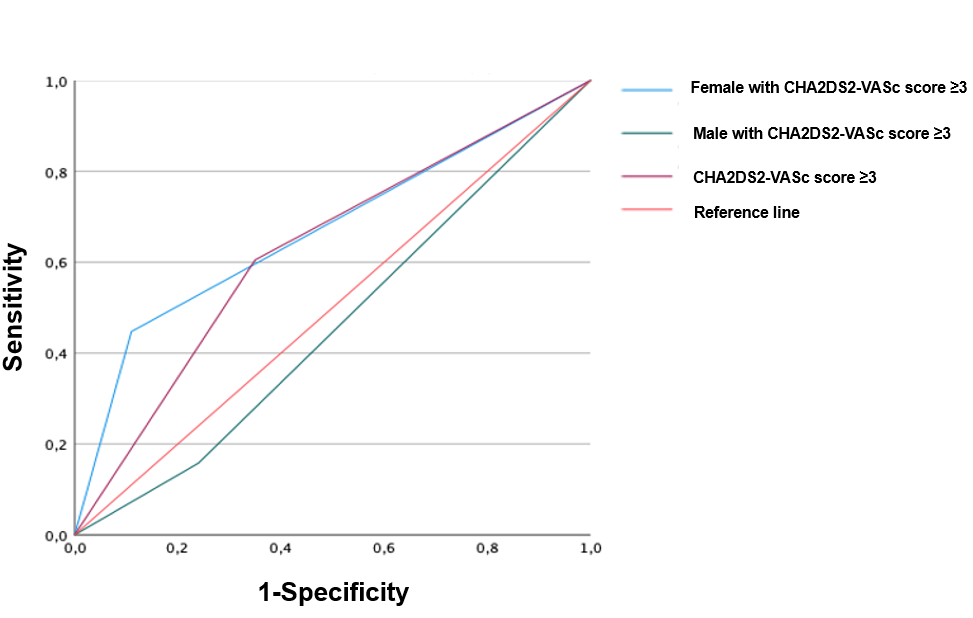


AUC 67 [56-78] %, p= 0.002: sensitivity 44.7%, specificity 89%.

AUC 46 [35-56] %, p= 0.457: sensitivity 15.8%, specificity 76%.

AUC 63 [52-73] %, p= 0.021: sensitivity 60.5%, specificity 65%.

Data are presented as median (25^th^-75^th^ percentile) for quantitative variables. A two-tailed p value of <0.05 was considered significant. *LA = left atrial; LVZ = low voltage zone; AUC = area under the curve.*

**Supplemental Figure 3.** Receiver operating charecteristic curves of LA indexed volume for the prediction of LA LVZ (cut-off: ≥5%).


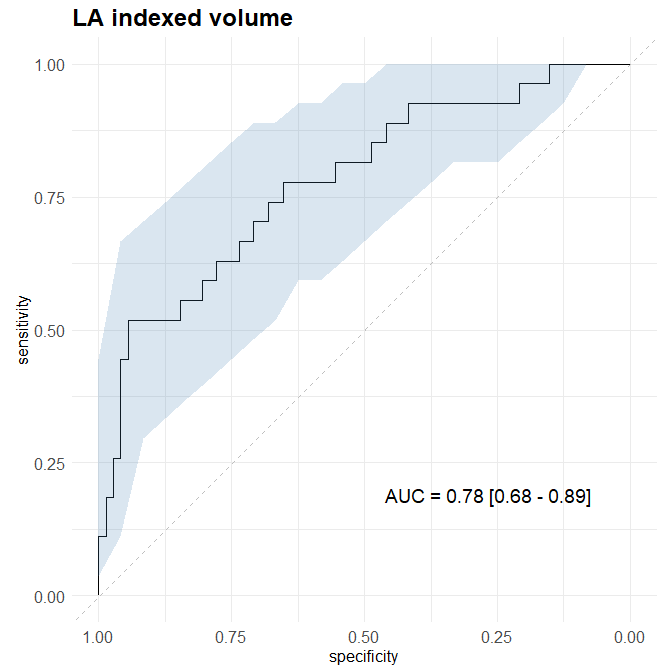


Data are presented as median (25^th^-75^th^ percentile) for quantitative variables. A two-tailed p value of <0.05 was considered significant. *LA = left atrial; LVZ = low voltage zone; AUC =* *area under the curve.*
